# “Crisis” or “opportunity”? COVID-19 pandemic's impact on environmentally sound invention efficiency in China

**DOI:** 10.3389/fpubh.2022.1102680

**Published:** 2023-01-20

**Authors:** Xuan Wei, Ranran Liu, Zhouzhou Lin

**Affiliations:** ^1^School of Statistics and Mathematics, Shandong University of Finance and Economics, Jinan, China; ^2^School of Technology and Business, Shandong Management University, Jinan, China; ^3^School of Politics and Public Administration, Soochow University, Suzhou, China

**Keywords:** COVID-19 pandemic, environmentally sound invention (ESI), ESI efficiency, environmental sound technologies (ESTs), Slack-Based Measure (SBM), spatial Tobit, stochastic frontier analysis (SFA)

## Abstract

**Introduction:**

The environmentally sound invention (ESI) is a “bridge” between environmental sound technologies (ESTs) and green productions. This study investigates the COVID-19 pandemic's impact on ESI efficiency using a multi-methods model in three stages.

**Methods:**

The ESI efficiency is measured using the Slack-Based Measure (SBM) method in the first stage. By excluding the environmental effect of the pandemic on each province using the stochastic frontier analysis (SFA) model's results in the second stage, this study compares the ESI efficiency change with or without the influence of the pandemic in the third stage.

**Results:**

The results show that the pandemic can be a “crisis” in the short term, but an “opportunity” in the long term. First, the SBM efficiency results in the first stage show a decrease in the number of the average efficient provinces in which the pandemic is more severe during 2020-2021. Second, results of the spatial Tobit and SFA models provide evidence that the COVID-19 pandemic negatively impacts the ESI efficiency during 2020, this impact is decreasing in 2021, and this impact has a spatial diffusion effect.

**Discussion:**

Based on these results, this study discussed the theoretical and political implications. This paper enriches the knowledge of ESTs research and development by proposing a three-stage approach with multi-methods to investigate the influence of the pandemic's impact on ESI efficiency.

## 1. Introduction

To improve environmental performance and against climate change, Environmentally Sound Technology (EST) was defined and became a major component of international collaborations since the Rio Summit in 1992 (1). In September 2020, China put forward the dual-carbon goals, including carbon peaking before 2030 and carbon neutrality before 2060. One of the operational guidelines in the Chinese action plan to reach such goals is leveraging the government's and the market's strengths (2). The operational guideline aims to accelerate the low-carbon technological revolution, emphasizing the importance of ESTs.

**Figure 1:**
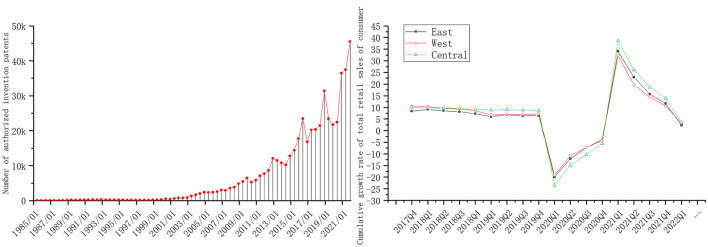
The trend of EST and the economy under the COVID-19.

China has been paying attention to ESTs and their innovation for several years, and their origin and development can be seen in a series of studies since the 2000s (3). It can also be seen from the left part of Figure 1 that the number of Environmentally Sound Invention (ESI) patents granted in China has shown an exponential growth trend since the 1990s. It is of great significance to ensure the steady improvement of ESTs for an eco-friendly development pattern (4).

However, in the dual-carbon goals scenario, the COVID-19 pandemic, a major public health emergency that began in December 2019, has widely affected many aspects of socio-economic development (e.g., the trend of the cumulative growth rate of retail sales of consumers in China shows in the right part of Figure 1). From a theoretical perspective, this pandemic contains two aspects: “crisis” and “opportunity”, i.e., the occurrence of the pandemic generates forces that hinder development and promote development simultaneously. In terms of “crisis”, the pandemic has resulted in the accelerated decoupling of China from global supply chains and the relocation of strategic manufacturing out of China (5). In terms of “opportunity”, the large-scale government interventions to cope with the pandemic are expected to give rise to an opportunity for a green recovery (6).

The ESTs listed by the United Nations Framework Convention on Climate Change (UNFCCC) (7) provided a reference to measure the quantity information of the ESI and its efficiency. However, the aspects of “crisis” or “opportunity” that dominate the impact on the ESI efficiency still lack empirical evidence. Therefore, the main goals of this study are as follows. First, this study needs to measure the ESI efficiency before and after the COVID-19 pandemic. ESI patent data are used as the output indicators. The rationale for this measure is the good nature of invention patents. I.e., the “China Patent Law (Revised in 2020)” stipulates that “the invention granted with the patent right shall be novel, creative and practical”, which implies that the patent examination system has a good function of checking technological innovation. Past empirical studies have also shown the effectiveness of measuring innovation through patent statistics (8). Second, this study aims to measure the different impacts of the COVID-19 pandemic on the quantity and efficiency of the ESI. Third, this study tries to answer the question of whether the direction and significance of the efficiency change will be different without the COVID-19 pandemic.

The main contributions of this study are as follows.

First, ESI and ESI efficiency are new notions in academic research, although they are familiar in practice. A provincial level's ESI efficiency based on the Slack-Based measure (SBM) method is constructed in this study. This study uses three types of regional ESI patent data, including firms, universities, and firm-university collaborations (FUCs), as the output of ESI and uses the SBM method to calculate the ESI efficiency from 2013 to 2021. Therefore, the efficiency difference before and after the pandemic in different regions can be compared preliminarily.

Second, a multi-methods model in three stages to examine the impact of COVID-19 on ESI efficiency is constructed. In particular, this study establishes the spatial Tobit and SFA models to test the direct and indirect impacts of the COVID-19 pandemic on ESI output and ESI efficiency. The Tobit model captures the pandemic's impact on the ESI efficiency. The SFA model captures the impact on the potential increase of the output of ESI. This study also distinguishes the pandemic's heterogeneous impact on the ESI efficiency of firms, universities, and FUCs.

Third, this study provides empirical evidence that the pandemic can be a “crisis” in the short term but an “opportunity” in the long term for the ESI efficiency. This research uses the environmental condition adjusting technology to calculate the ESI efficiency under the same environmental condition and makes a comparative analysis with the efficiency in the first stage.

The remainder of this paper is organized as follows. Section 2 gives a literature review including the theoretical background of ESI and its efficiency. The research design, material, and methods are described in Section 3. Section 4 is the empirical findings. Following the discussion in Section 5 to explore the theoretical and practical implications of this study. We make the conclusions and practical implications in Section 6 and practical implications and give limitations and future research in Section 7.

## 2. Literature review

### 2.1. Theoretical background of ESI and its efficiency

**Figure 2:**
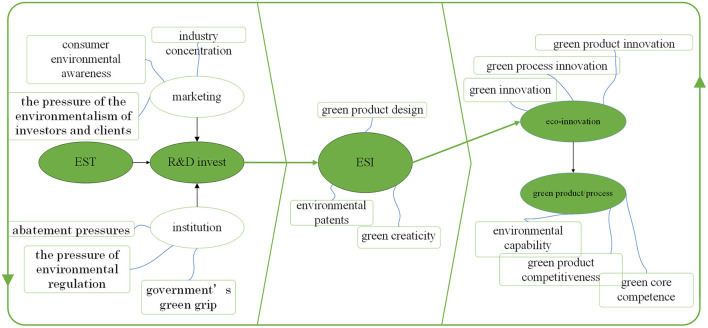
Concepts and their relationships related to ESI.

From a narrow sense, inventions are new or improved devices', products', or processes of systems' ideas, sketches, or models (9). From a broad sense, inventions are the production of knowledge (10). Although the invention is not unfamiliar in both practice and academic research documents (11). Environmentally sound invention (ESI) is a new notion in academic research to our knowledge. Figure 2 shows key concepts and their relationships related to ESI according to previous research. It is important because ESI can be a “bridge” between EST and green products/processes.

The right part of Figure 2 describes that ESI is a part of the process of green innovation (11). There are some different concepts to explain similar innovation processes toward sustainable development. Two representative concepts are eco-innovation (12) and environmental innovation (13). It can be divided into green product innovation (14) and green process innovation (15). Innovation can be successful or unsuccessful (16). Therefore, there is a capability view that suggests that ESI's producers should enhance their related capabilities such as environmental capability (17) and green core competence (18).

The left part of Figure 2 is the source of ESI. Studies on EST began in about 1990s after the Agenda 21 defined it (19). R&D activities based on internal and external EST accumulation have seemed as the direct source of ESI, i.e., the so-called technology push (20). Porter and Van der Linde's (21) foundational work clarifies the relationship between environmental goals and industrial performance, i.e., environmental regulation can reduce innovation offsets. Therefore, institutional factors can influence R&D activities on ESI. Related connotations include abatement pressure (22), pressure of environmental regulation (23), and government's greengrip (24). Another driving force of R&D activities on ESI is marketing, i.e., the so-called demand-pull (20). Related connotations include industry concentration (25), consumer environmental awareness (26), and pressure of the environmentalism (27).

Then, the connotation of ESI in the center of Figure 2 and how to measure its efficiency can be clearer. In Figure 2, from the left part to the central part, ESI is the output of R&D activity which is influenced by marketing and institution. From the central part to the right part, ESI is the source of eco-innovation and green products. There are several similar concepts compared with ESI, including green product design (28), environmental patents (29), and green creativity (30). In sum, for measuring the ESI efficiency, the input and influencing factors can be found in studies of R&D activities on the base of EST, marketing, and institution regulation. The output factors can be found in studies of eco-innovation and environmental capabilities.

### 2.2. Impact of the COVID-19 pandemic on ESI and its efficiency

When the innovation theory was put forward, Schumpeter (9) had already clarified that the crisis can breed innovation and development in the economic cycle. Moreover, although the “crisis” Schumpeter mentioned belongs to the economic category, it is defined as the result of external things acting on the economic field and causing interference. Therefore, external disturbances can play a significant role in innovation-driven economic cycles. In environmental and sustainable studies, other connotations of external disturbances that have been defined include environmental uncertainty (31) and environmental dynamism (32). On the one hand, major public emergencies may disrupt innovation in the previous economic process (33). On the other hand, public emergencies can breed new innovations through demand-pull, supply-push (34), or the emergence of crowd wisdom (35).

The empirical research results support this contradictory theory. On the one hand, from the perspective of the input of ESI, some empirical studies are optimistic about the impact of the COVID-19 pandemic on ESI. For example, previous research has drawn attention to the environmental pollutant treatment technology (36) and the food supply chain's waste problem (37) due to the pandemic and expects substantial progress in related technologies (38). The investment in smart city projects in China during the COVID-19 pandemic reduced the infection rate and promoted the innovative development of digital industries and urban sustainable technologies (39). Studies in Brazil and Portugal show that the pandemic has significantly enhanced the awareness of environmental protection and sustainable consumption among the residents of Baby Boomers and Generations X and Y (40).

On the other hand, from the perspective of the output of ESI, there is also evidence highlighting the negative impact of the pandemic on ESI. For example, a survey of 526 companies in Norway showed that the average level of firms' adoption of environmental innovations had dropped significantly due to the COVID-19 pandemic (41). The COVID-19 pandemic has affected the implementation of plastic reduction policies in Europe and around the world, thus leading to an increase in plastic waste, which harms the environment and human health (42). The macroeconomic blockade effect caused by the pandemic has brought many adverse effects on the innovation of SMEs (43).

It can be noticed that topics of the optimistic studies, including promotion in EST, investment in smart city projects, environmental protection awareness, and sustainable consumption, are all input factors for ESI. Topics of pessimistic studies, including firms' adoption of environmental innovations, increase in plastic waste, and negative effects on SMEs' innovation, are all output factors for ESI.

According to this background, although there is still no previous study research on the effect of the COVID-19 pandemic on ESI efficiency, this study put forward the following hypotheses.

HYPOTHESIS 1. *The COVID-19 pandemic has a negative effect on regions' ESI efficiency*.

Moreover, the spatial effects of the COVID-19 pandemic on human activities may result in changes in ESI efficiency across regions. For example, Chen et al. (44) found that the lockdown policy because of the pandemic had resulted in a decrease in the flow of goods and services between cities. Huggins and Thompson (45) argued that the pandemic is likely to heighten rather than slow down the trend that more spatially distributed patterns of entrepreneurial innovation are emerging across a wider range of cities and regions. Korkmaz et al. (46) revealed that the response to the pandemic had caused education inequalities across regions. Dannenberg et al. (47) believed that the increase in online trade which was led by the pandemic has changed the trend of the spatial economics of innovation. Therefore, this study put forward the following hypotheses.

HYPOTHESIS 2. *The COVID-19 pandemic has a significant spatial effect on ESI efficiency*.

## 3. Research design, material, and methods

### 3.1. Research design

This study uses a multi-methods model in three stages to examine the impact of COVID-19 on ESI efficiency. There is a progressive foreshadowing between different methods at each stage and a relationship of mutual robustness. In the first stage, this study uses the SBM method to calculate the non-radial efficiency score of ESI. Whether the efficiency scores in different provinces have significant statistical changes before and after the pandemic outbreak was preliminarily analyzed. In the second stage, this study tests the significance of the impact of the pandemic on efficiency using spatial Tobit regression. And we test the significance of the influence of the pandemic on the potential increase of different types of ESI output using the spatial SFA model. In the third stage, when the significant impact is determined in the second stage, we use the environmental effect adjustment technology of the three-stage DEA (48) to adjust the environmental conditions faced by each region to the same level and analyze the efficiency changes in each region compared with the first stage.

### 3.2. Green technological innovation efficiency

This study uses the SBM method to measure the ESI efficiency. On the one hand, this method overcomes the disadvantage that the input and output of each decision-making unit (DMU) can only be proportionally expanded or reduced in the traditional radial DEA model (Data Envelopment Analysis). And on the other hand, it can directly measure the slack value of each variable (49), which is convenient for establishing the subsequent SFA model. Assuming that the input vector of the ESI of *k*th (*k*∈1,…,*n*) DMU is $X_{k}={\left[x_{1},x_{2},\ldots ,x_{m}\right]}^{T},(m\geq 1),$, the output vector is $Y_{k}={\left[y_{1},y_{2},\ldots ,y_{s}\right]}^{T},(s\geq 1),$, define the set of production possibilities as follows:


$$P=\left\{\left(x,y\right)|x\geq \sum_{k=1}^{n}X_{k}\lambda_{k},y\geq \sum_{k=1}^{n}Y_{k}\lambda_{k},\lambda_{k}\geq 0\right\}$$

where λ_*k*_ is the decision coefficient. Then for the DMU **P**_0_ = {(**x**_0_, **y**_0_)}, the formula for calculating the ESI efficiency value is:


$$\begin{array}{l} \rho_{0}=\min\frac{1-\frac{1}{m}\sum_{i=1}^{m}\frac{s_{i0}^{-}}{x_{i0}}}{1+\frac{1}{s}\sum_{r=1}^{s}\frac{s_{r0}^{+}}{y_{r0}}}\\ \;s.t.\{\begin{array}{c} x_{0}=\sum_{k=1}^{n}X_{k}\lambda_{k}+s_{0}^{-}\\ y_{0}=\sum_{k=1}^{n}Y_{k}\lambda_{k}-s_{0}^{+}\\ s_{0}^{-}\geq 0,s_{0}^{+}\geq 0,\lambda_{k}\geq 0 \end{array} \end{array}$$

where ρ_0_ is the DMU's ESI efficiency score of **P**_0_ = {(**x**_0_, **y**_0_)}. **x**_0_ is the vector of the input variable value, *x*_*i*0_ is its *i-*th dimension. **y**_0_ is the vector of the output variable value, *y*_*r*0_ is its *r-*th dimension. $s_{0}^{-}$ is the vector of the input variables' slack or the so-called “potential decrease”, $s_{i0}^{-}$ is its *i-*th dimension. $s_{0}^{-}$ is the vector of the slack or potential increase of the output variables, and $s_{r0}^{+}$ is its *r-*th dimension.

### 3.3. Model

#### 3.3.1. Spatial Tobit model

The value interval of the ESI efficiency is [0,1], which is not a normal distribution and does not meet the fitting model of the general least squares method. Therefore, to study the impact of the COVID-19 pandemic on the ESI efficiency, this study adopts the Tobit regression method. At the same time, considering the possible spatial spillover effects of the ESI, pandemic, and other variables, referring to the research of Li and Hong (50), this study constructs the following spatial Tobit regression model with ESI efficiency as the explained variable:


$$\begin{array}{l} \text{ρ=λWρ+}\left(\text{COVID,CONTROL}\right)\text{β}\\ \text{ +W}\left(\text{COVID,CONTROL}\right)\text{δ+μ}\\ \text{ μ=λWμ+ε} \end{array}$$

where ρ represents the vector of the efficiency scores. **W** is a spatial weight matrix. The spread of the pandemic has a natural correlation with geographic location. Therefore, this study uses geographic adjacency to establish the spatial weight matrix. λ**Wρ** represents the spatial effect of the efficiency of other regions. (**COVID**, **CONTROL**) is the block matrix composed of the CIVID-19 variable and control variables, **β** is the coefficient matrix of the block matrix. **W**(**COVID**, **CONTROL**)**δ** represents the spatial effect of the independent variable and control variables in other regions. **μ** represents the spatial effect of random disturbance, **ε** is the random error term. When **μ** = 0, the model is a spatial independent variable lag model, when **μ** = 0 and **δ** = 0, the model is a spatial autoregressive model, and when λ = 0 and **δ** = 0, the model is a spatial error model.

#### 3.3.2. SFA model

This study refers to the three-stage DEA model constructed by Fried et al. (48) to test the impact of the COVID-19 pandemic on the slack of ESI output and to analyze the efficiency changes of each DMU that has adjusted the pandemic environment to the same level.

Taking the slack of the output variables in the first-stage SBM model as the dependent variable, the SFA model is as follows:


$$s_{r}^{+}=f\left(COVID,CONTROL;\beta_{c},\beta_{con}\right)+v_{r}+\mu_{r}$$

where $s_{r}^{+}$ is the DMU's slack of the *r-*th output variable. *COVID* and **CONTROL** are the environmental conditions faced by the DMU. β_*c*_ and **β**_*con*_ are the parameters to be estimated. *v*_*r*_+μ_*r*_ represents the mixed error, where $v_{r}\sim N\left(0,\sigma_{\nu }^{2}\right)$ represents the effect of random factors, μ_*r*_≥0 represents the effect of management inefficiency. Assuming that $\mu_{r}\sim N^{+}\left(\mu^{s},\sigma_{\mu }^{2}\right)$, *v*_*r*_ and μ_*r*_ are independent of each other.

#### 3.3.3. Adjusting of environmental effects

Based on the results of the SFA model, the equation for slack adjustment is as follows:


$$\begin{array}{l} x_{r}^{A}=x_{r}+[\max\left\{\left(COVID,CONTROL\right){\left(\hat{\beta_{c}},\beta \hat{{}_{con}}\right)}^{T}\right\}\\ \;-\left(COVID,CONTROL\right){\left(\hat{\beta_{c}},\hat{\beta_{con}}\right)}^{T}]+\left[\max\left\{\hat{v_{r}}\right\}-\hat{v_{r}}\right] \end{array}$$

where *x*_*r*_ and $x_{r}^{A}$ are the values of the *r-*th output before and after adjustment, respectively. Therefore, the adjusted output value can be used to evaluate and compare the green innovation efficiency with the results in the first stage.

### 3.4. Data

#### 3.4.1. Input-output variables in SBM

The input-output variables in this study are shown in Table 1. The input factors on the demand side are usually difficult to measure (51). Therefore, this study mainly considers the input variables on the supply side. The input variables include government R&D (Research and Development) expenditure, R&D expenditure of industrial firms above the designated size, R&D personnel of industrial firms above the designated size, and the number of senior full-time teachers in universities (52). The output variable is selected according to the type of inventors' organizations (53), including the number of ESI patent applications of firms, universities, and FUCs. The reason for choosing the number of ESI patent applications is that invention patents have higher requirements for novelty and creativity than utility model and design patents, and the measurement of technological innovation is more accurate (54). On the other hand, the number of applications has better timeliness and stability than the number of authorized patents. The data source of the ESI patent applications is the Patsnap Database (https://www.patsnap.com/). We screened the ESI patents using the International *Green Patent Inventory* published by the World Intellectual Property Organization (https://www.wipo.int/classifications/ipc/green-inventory/). The patent data generally have the problem of duplicate data of the same application with different publication numbers. This study deletes duplication according to the same application documents and counts them according to the oldest application date. The time horizon for the output variable is 2013–2021 (data collection time is May 2022, so the effect of not the first disclosure of patents filed in 2021 can be ignored). The data source of the input variable is the website of the National Bureau of Statistics, and the time is 2012–2020. That is, a 1-year lag period is set between the input variable and the output variable. Due to the problem of missing data and abnormal values, provinces data from Tibet, Qinghai, Hainan, Hubei, Hong Kong, Taiwan, and Macau are not included.

**Table 1:** Input-output variables of SBM model and data sources (*N* = 243).

**Variables**		**Mean**	**Min**	**Max**	**Data source**
Input	Government R&D expenditure	141.83	9.61	1,168.79	
	R&D expenditure of industrial firms above designated size	394.20	14.37	2,499.953	
	Number of R&D personnel in industrial firms above designated size	9.93	0.42	70.00	
	Number of senior full-time teachers in universities	0.71	0.09	2.28	
Output	Number of universities' ESI patent applications	777.03	9.00	5,818.00	
	Number of firms' ESI patent applications	2,490.33	37.00	21,164.00	
	Number of FUCs' ESI patent applications	113.59	1.00	1,959.00	

State Intellectual Property Office of China; Patsnap Database. The units of government R&D expenditure, R&D expenditure of industrial firms above designated size, R&D personnel of industrial firms above designated size, and the number of senior full-time teachers in universities are 100 million yuan, 100 million yuan, 10,000 people, and 10,000 people, respectively; the unit of ESI applications is pieces.

#### 3.4.2. Independent and control variables

Based on previous research on the severity of the COVID-19 pandemic (55). This study uses the number of confirmed COVID-19 cases (*confirmed*) as the primary explanatory variable. The data on the number of confirmed cases comes from the pandemic monitoring data of *DingXiangYiSheng* (https://ncov.dxy.cn/ncovh5/view/pneumonia). The period of the data is 2020–2021. At the same time, this study sets a series of variables that impact green technological innovation efficiency as control variables. The main theoretical basis is the supply-push theory, demand-pull theory, and Porter hypothesis which have been mentioned in Section Theoretical background of ESI and its efficiency. The indicators include GDP (Gross Domestic Product, supply-push factor) (56), total investment in industrial pollution control (poluinvest) (demand-pull factor) (57), industrial water consumption (usewater) (demand-pull factor) (58), and dummy variables set for SO_2_'s or carbon's trading market's pilot provinces (environmental regulatory factors) (59). The data source is the website of the National Bureau of Statistics of China. The period of the data is 2012–2020, i.e., a one-year lag is set for the impact of control variables on the efficiency of green technological innovation and potential output increase. The descriptive statistics of the data are shown in Table 2.

**Table 2:** Descriptive statistics of variables.

**Variable type**		**Symbol**	***N***	**Mean**	**SD**	**Min**	**Max**
Independent		*Confirmed*	54	139.10	355.90	0.000	2,046.00
Control	Supply-push	*GDP*	243	2.95	2.30	0.23	12.44
	Demand-pull	*Poluinvest*	243	25.05	22.73	512.20	141.65
		*Usewater*	243	44.10	47.28	3.00	255.20
	Environmental regular	*Shidian*	27	0.43	0.50	0.00	1.00

The units of confirm, GDP, poluinvest, and usewater are people, trillion yuan, billion yuan, and billion cubic meters, respectively.

## 4. Empirical findings

### 4.1. Efficiency evaluation results

Based on each province's evaluation result of ESI efficiency shown in Table 3. Table 4 shows a change in the number of efficient DMUs from 2013 to 2021. From the following two perspectives, it can be preliminarily judged that the pandemic has negatively affected the ESI efficiency.

**Table 3:** Provinces' ESI efficiency and means during 2013–2021.

**DMU**	**Zone**	**2013**	**2014**	**2015**	**2016**	**2017**	**2018**	**2019**	**2020**	**2021**
Anhui	HL	0.37	0.74	**1.00**	**1.00**	**1.00**	**1.00**	**1.00**	0.54	0.42
Beijing	HL	**1.00**	**1.00**	**1.00**	**1.00**	**1.00**	**1.00**	**1.00**	**1.00**	**1.00**
Fujian	HH	0.21	0.17	0.27	0.30	0.39	0.40	0.34	0.28	0.30
Gansu	LH	0.46	0.33	0.36	0.43	0.56	0.48	0.70	0.48	0.59
Guangdong	LH	0.47	0.38	0.51	0.49	**1.00**	**1.00**	**1.00**	**1.00**	**1.00**
Guangxi	LL	0.47	0.50	0.64	**1.00**	**1.00**	0.50	0.42	0.39	0.41
Guizhou	LH	0.32	0.45	0.30	0.30	0.45	0.37	0.23	0.23	0.22
Hebei	LL	0.13	0.14	0.13	0.29	0.24	0.29	0.31	0.25	0.72
Henan	LH	0.19	0.19	0.25	0.30	0.40	0.36	0.34	0.23	0.27
Heilongjiang	LL	0.49	0.48	**1.00**	0.43	**1.00**	**1.00**	**1.00**	**1.00**	**1.00**
Hunan	LH	0.22	0.32	0.34	0.35	0.53	0.43	0.47	0.31	0.34
Jilin	HL	0.32	0.25	0.36	0.31	0.47	0.45	0.71	0.56	0.68
Jiangsu	HH	**1.00**	**1.00**	**1.00**	**1.00**	**1.00**	**1.00**	**1.00**	**1.00**	**1.00**
Jiangxi	HH	0.17	0.17	0.15	0.22	0.32	0.27	0.22	0.20	0.18
Liaoning	LH	0.38	0.33	0.34	0.46	**1.00**	**1.00**	**1.00**	0.50	0.51
Neimenggu	HL	0.10	0.07	0.12	0.16	0.13	0.21	0.26	0.25	0.47
Ningxia	HL	0.70	0.31	0.28	0.19	0.24	0.26	0.21	0.26	0.44
Shandong	LL	0.37	0.43	0.44	0.40	0.47	0.47	0.37	0.33	0.34
Shanxi	HH	0.20	0.12	0.15	0.23	0.34	0.39	0.32	0.31	0.32
Shannxi	LL	**1.00**	**1.00**	0.60	0.45	0.82	0.97	**1.00**	**1.00**	**1.00**
Shanghai	LH	**1.00**	**1.00**	0.65	**1.00**	0.67	0.56	**1.00**	**1.00**	**1.00**
Sichuan	LH	**1.00**	0.41	0.86	0.69	**1.00**	**1.00**	0.46	0.35	0.42
Tianjin	HL	0.80	0.62	0.84	0.64	**1.00**	**1.00**	**1.00**	0.56	0.54
Xinjiang	HL	0.23	0.17	0.23	0.29	0.26	0.27	0.37	0.34	0.49
Yunnan	HH	0.51	0.65	0.73	0.68	0.70	**1.00**	0.65	0.49	0.54
Zhejiang	HL	0.30	0.28	0.38	0.37	0.55	0.58	0.55	**1.00**	**1.00**
Chongqing	HH	0.34	0.47	**1.00**	0.42	0.44	0.47	**1.00**	0.47	0.54

The bold values are corresponding province is efficient.

**Table 4:** Number of efficient DMUs during 2013–2021.

		**2013**	**2014**	**2015**	**2016**	**2017**	**2018**	**2019**	**2020**	**2021**
Number of efficient DMUs		5	4	5	5	9	9	10	7	7
Average rank	HL	11	13	11	12	14	9	7	7	7
	HH	13	12	11	12	16	8	8	11	12
	LH	9	11	11	8	10	6	6	10	10
	LL	9	9	9	9	11	6	7	8	7

First, from the number of provinces at the frontier each year, the ESI efficiency from 2013 to 2021 can be roughly divided into three stages. The first stage is from 2013 to 2016, and the number of provinces at the frontier is 4–5 each year. The second stage is from 2017 to 2019, and the number of provinces at the frontier is 9–10 each year. The third stage is from 2020 to 2021, and the number of provinces at the frontier is 7 each year.

**Figure 3:**
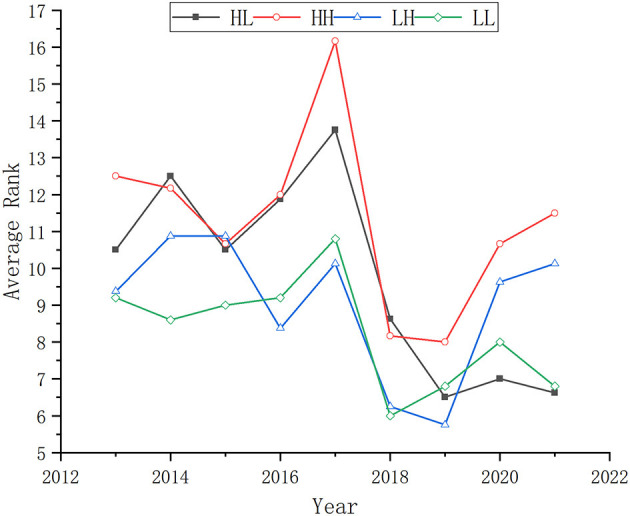
Variation of the average rank of ESI efficiency score in different zones.

Second, this study divides different provinces into four types based on the severity of the pandemic. When the number of confirmed cases in a province is in the first half of all provinces in a year, the severity is defined as “High”. Otherwise, the severity is defined as “Low”. There are four different types of provinces: High in 2020 and Low in 2021 (HL), High in 2020 and High in 2021 (HH), Low in 2020 and High in 2021 (LH), Low in 2020 and Low in 2021 (LL). This study analyzes the average rank of ESI efficiency scores of different types of provinces. The more efficient DMUs, the little the average rank of ESI efficiency scores. Its trend chart is shown in Figure 3. It can be seen from Figure 3 that there is a peak of the average rank of all types of provinces in 2017. And the average rank of the four types of provinces is at the lowest point in 2019. The average rank of the HH provinces is the lowest in 2020 and 2021. While the leader of the rank is the HL provinces in 2020 and LL provinces in 2021. Moreover, the rank of HH and LH provinces has a declining trend, and the rank of HL and LL provinces has an increasing trend.

These efficiency rank results show an abnormal decline in the number of efficient DMUs in 2020, which is synchronized with the outbreak of the COVID-19 pandemic. The trend of the average rank of different types of provinces also shows a correlation between the pandemic and ESI efficiency. Therefore, these results preliminarily prove that the pandemic has a negative effect on ESI efficiency. However, it is uncertain whether there is an explanation-to-explained relationship between the outbreak's severity and this phenomenon. Therefore, more inspection is necessary.

### 4.2. Spatial Tobit model selection and results

It is necessary to determine the form of the spatial model before estimating it. The Moran's I is calculated for each variable; the results are shown in Table 5. From Table 5, the Moran's I of the dependent variables, including the ESI efficiency score and the total number of ESI patent applications, are both nonsignificant. Therefore, the spatial lag model is initially excluded. Among the independent variables, the *p*-value of the Moran's I of confirmed cases is significant in 2020 and nonsignificant in 2021. The pandemic outbreak in 2020 may make a more significant spatial impact, and in 2021, the effective control policies curb the spread between regions. The *p*-values of the Moran's I for the control variables are all significant. It implies that it is necessary to consider the spatial effect of independent variables and control variables.

**Table 5:** Moran's I test results for each variable.

**Variable**		**Year**	**Moran's I**	***E*(*I*)**	**sd(*I*)**	***t***	***p*-value**
Dependent	Total invention patent applications	2021	0.12	−0.04	0.12	1.42	0.17
		2020	0.16	−0.04	0.12	1.69	0.10^*^
	Efficiency score	2021	0.01	−0.04	0.12	0.38	0.71
		2020	0.02	−0.04	0.12	0.46	0.65
Independent	*Confirmed*	2021	−0.16	−0.04	0.12	−1.01	1.68
		2020	0.17	−0.04	0.12	1.75	0.09^*^
Control	*GDP*	2020	0.26	−0.04	0.12	2.57	0.02^*^
		2019	0.26	−0.04	0.12	2.54	0.02^*^
	*Usewater*	2020	0.26	−0.04	0.08	3.57	0.00^***^
		2019	0.26	−0.04	0.09	3.25	0.00^**^
	*Shidian*	2020	0.27	−0.04	0.12	2.64	0.01^**^
		2019	0.42	−0.04	0.11	4.14	0.00^***^

2-tail test; ^*^, ^**^, ^***^ indicate 10, 1, and 0.1% significance, respectively.

Table 6 further shows the judgment and selection of the spatial econometric model. The main judgment indicators include Moran's I, Lagrangian multiplier (LM), and Robust Lagrangian multiplier (Robust LM). It can be found that the fitting results of the spatial error model and the spatial lag model of the four different dependent variables are all poor. Additionally, considering that the *p*-values of the efficiency in Table 5 are not significant, this study excluded the spatial error model and the spatial lag model. The spatial independent variable lag model is used without spatial error and spatial lag.

**Table 6:** Spatial measurement model selection.

**Dependent variable**		**Spatial error**			**Spatial lag**	
		**Moran's I**	**LM**	**Robust LM**	**LM**	**Robust LM**
ESI efficiency in 2021	Statistic	0.47	0.24	0.63	0.68	1.07
	*p-*value	0.64	0.63	0.43	0.41	0.30
ESI efficiency in 2020	Statistic	0.65	0.05	0.36	0.27	0.57
	*p-*value	0.51	0.82	0.55	0.61	0.45
Total ESI patent applications in 2021	Statistic	0.44	0.36	0.71	0.07	0.43
	*p-*value	0.66	0.55	0.40	0.79	0.52
Total ESI patent applications in 2020	Statistic	0.61	0.14	0.35	0.02	0.22
	*p-*value	0.54	0.70	0.55	0.90	0.64

In a spatial independent variable lag model without spatial error and dependent variable lag effect, the classical linear model's estimation and statistical inference methods are unbiased. Therefore, special estimation and statistical inference methods are unnecessary (60). Table 7 shows the regression results with spatial effects of independent variables. The dependent variables of Model (1) and Model (2) are the numbers of ESI patent applications in 2021 and 2020, respectively. The dependent variables of Model (3) and Model (4) are ESI efficiency scores measured in 2021 and 2020, respectively.

**Table 7:** Spatial Tobit regression results.

**Variable**	**Total invention patent applications in 2021**	**Total invention patent applications in 2020**	**Efficiency in 2021**	**Efficiency in 2020**
	**(1)**	**(2)**	**(3)**	**(4)**
*Confirmed* in 2021	−0.8488 (−0.4798)		−0.0001^*^ (2.0543)	
*Confirmed* in 2020	−1.8555 (−0.5071)	−1105.9037 (−0.6491)	−0.0000 (−0.1411)	0.0001 (0.8938)
W*confirmed* in 2020	0.1496 (0.0655)	−0.5565 (−0.2842)	0.0001^*^ (1.8437)	0.0001 (1.6133)
W*confirmed* in 2021	−0.6570 (−0.6049)		−0.0000 (−0.4866)	
Control variable and its spatial effect	Yes	Yes	Yes	Yes
One-year lag of the dependent variable	Yes	Yes	Yes	Yes
Var(e.te2021)			0.0067^**^ (3.1117)	
Var(e.te2020)				0.0158^**^ (2.9937)
*N*	27	27	27	27
Adj. *R*^2^	0.6760	0.7493		
Log-likelihood	−255.5812	−253.9254	20.3231	8.9262

Standard errors in parentheses; ^*^, ^**^ indicate 10% and 1% significance respectively. The logarithm of the independent and control variables is processed.

From the results of Model (1) and Model (2), the number of confirmed cases has no significant impact on the output of ESI patents in the current and next years. It shows that the direct impact of the COVID-19 pandemic on ESI is not significant. From Model (3) and Model (4), the number of confirmed cases has no significant impact on ESI efficiency in 2020, but the impact of the confirmed cases on ESI efficiency in 2021 is significantly negative. In addition, the spatial effect of the confirmed cases in 2020 on ESI efficiency 2020 is significantly positive. It implies at the first year of the outbreak, although the COVID-19 pandemic has no significant direct impact on ESI efficiency, and a province's ESI efficiency will increase as the number of confirmed cases in the surrounding region increases. These results are accordant with the results shown in Table 4 that the rank of the ESI efficiency of provinces with fewer confirmed cases is relatively higher than provinces with severe pandemic environments. This evidence further supports the hypothesis that the pandemic has a negative effect on ESI efficiency.

### 4.3. Spatial SFA model results

Considering the spatial autocorrelation, the spatial effects of independent and control variables are incorporated into the SFA model. The results are shown in Table 8. The results include a total of 6 models. The dependent variables of Model (1)–Model (3) are the slack values of the output variables in the SBM model in 2020, i.e., the number of ESI patent applications of firms, universities, and FCUs, respectively. The dependent variables of Model (4)–Model (6) are the slack values of the output variables in the SBM model in 2021.

**Table 8:** Spatial SFA model results.

	**Output slacks in 2020**			**Output slacks in 2021**		
	**(1)**	**(2)**	**(3)**	**(4)**	**(5)**	**(6)**
*Confirmed* 2020	−0.6035^*^ (−1.7504)	−0.1244^*^ (−1.9235)	−0.0460 (−0.9644)	−0.4482 (−1.0069)	−0.0290 (−0.7927)	−0.1314 (−0.7920)
*Confirmed* 2021				−0.0890 (−0.4118)	−0.0211 (−1.1839)	0.0360 (0.4470)
W*confirmed*2020	−0.4777^*^ (−1.7474)	−0.153^***^ (−2.9864)	−0.0770^**^ (−2.0356)	−0.2276 (−0.7901)	−0.0523^**^ (−2.2072)	−0.0157 (−0.1465)
W*confirmed*2021				−0.0027 (−0.020)	0.0013 (0.1202)	−0.0641 (−1.2799)
Control	Yes	Yes	Yes	Yes	Yes	Yes
Spatial effect of Control	Yes	Yes	Yes	Yes	Yes	Yes
γ	0.148	0.249	0.283	0.145	0.331	0.201
*N*	27	27	27	27	27	27
Log-likelihood	−205.7206	−160.5329	−152.3359	−207.6133	−140.1647	−180.9738

Standard errors in parentheses; ^*^, ^**^, ^***^ indicate 10, 1, and 0.1% significance, respectively.

The results of Model (1)–Model (3) show that in 2020, the confirmed cases have a negative impact on the slack of ESI output. The spatial effect of the confirmed cases in 2020 also has a significant negative impact on the slack of ESI output. That indicates that a province with more confirmed cases may negatively impact the potential increase of ESI not only itself but also its surrounding regions. The results of Model (4)–Model (6) show that the impact of the confirmed cases is not significant on the slack of ESI output in 2021. Additionally, the spatial effect term of the confirmed cases in 2020 negatively impacts the slack values of universities in 2021. That indicates that the negative effect of the COVID-19 is decreasing in the second year. Moreover, the values of γ of Model (1)–(6) are between 0.1 and 0.4, indicating that environmental variables and statistical noise dominate the disturbance of insufficient output of ESI in firms. Therefore, it is judged that the continuous impact of the COVID-19 has created a more challenging external environment for EST innovation. These results indicate that a region with a more severe pandemic led to a more difficult environment for ESI output in 2020 and 2021 for both the region itself and regions surrounding it. Therefore, this evidence supports both hypothesis 1 and hypothesis 2.

### 4.4. Efficiency change after adjusting environmental conditions

The results of the mutual confirmation of the Tobit and SFA models show a significant negative influence of the COVID-19 pandemic on provincial ESI efficiency. This section further focuses on how each region's efficiency score would change if all regions' pandemic environments are adjusted to the same level. The comparison of the ESI efficiency score of the original SBM models with the post-adjusting models is shown in Table 9. Table 10 shows the change in the average rank of different types of provinces of the original and after adjusting the environment effect's efficiency measure.

**Table 9:** Comparison of original efficiency and efficiency after environmental effect adjustment.

**DMU**	**Zone**	**2020**		**2021**	
		**Original**	**AdjustCOVID-19**	**Original**	**AdjustCOVID-19**
Anhui	HL	0.54	**1.00**	0.42	**1.00**
Beijing	HL	**1.00**	**1.00**	**1.00**	**1.00**
Fujian	HH	0.28	0.50	0.30	0.49
Gansu	LH	0.48	**1.00**	0.59	**1.00**
Guangdong	LH	**1.00**	**1.00**	**1.00**	**1.00**
Guangxi	LL	0.39	0.61	0.41	0.51
Guizhou	LH	0.23	0.39	0.22	0.34
Hebei	LL	0.25	0.50	0.72	0.54
Henan	LH	0.23	0.30	0.27	0.29
Heilongjiang	LL	**1.00**	**1.00**	**1.00**	**1.00**
Hunan	LH	0.31	0.44	0.34	0.38
Jilin	HL	0.56	0.50	0.68	0.58
Jiangsu	HH	**1.00**	**1.00**	**1.00**	**1.00**
Jiangxi	HH	0.20	**1.00**	0.18	0.51
Liaoning	LH	0.50	0.25	0.51	0.36
Neimenggu	HL	0.25	**1.00**	0.47	**1.00**
Ningxia	HL	0.26	0.25	0.44	0.43
Shandong	LL	0.33	0.37	0.34	0.38
Shanxi	HH	0.31	0.48	0.32	0.39
Shannxi	LL	**1.00**	**1.00**	**1.00**	**1.00**
Shanghai	LH	**1.00**	**1.00**	**1.00**	**1.00**
Sichuan	LH	0.35	0.31	0.42	0.34
Tianjin	HL	0.56	0.40	0.54	0.47
Xinjiang	HL	0.34	0.31	0.49	0.47
Yunnan	HH	0.49	0.28	0.54	0.34
Zhejiang	HL	**1.00**	**1.00**	**1.00**	**1.00**
Chongqing	HH	0.47	0.53	0.54	0.54

The bold values are corresponding province is efficient.

**Table 10:** Comparison of original efficiency and efficiency after environmental effect adjustment.

		**2020**		**2021**	
		**Original**	**AdjustCOVID-19**	**Original**	**AdjustCOVID-19**
Number of efficient DMUs		7	11	7	10
Average rank	HL	7	5	7	4
	HH	11	5	12	8
	LH	10	8	10	10
	LL	8	5	7	5

**Figure 4:**
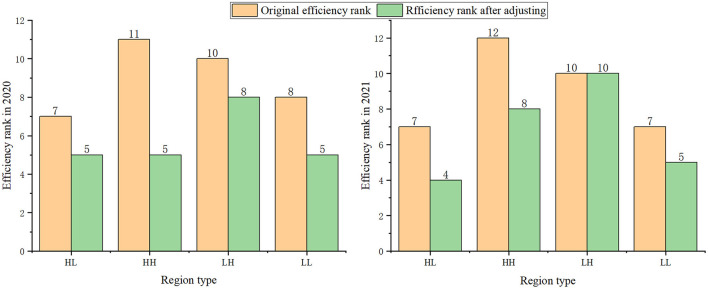
The impact of COVID-19 on green technological innovation in different regions.

When adjusting the pandemic environment faced by each province to the same level, the number of production frontiers in 2020–2021 increases significantly, which is consistent with the growth trend before the pandemic outbreak. This study further characterizes this trend in Figure 4. The change in the average rank of the efficiency scores of four types of provinces before and after the adjustment is shown in Figure 4. As seen from Figure 4, after adjusting, the average rank efficiency score of HH increase fastest among the four types in 2020. However, in 2021, the increase of the rank of HH and LH provinces is no longer faster than HL and LL provinces.

## 5. Discussion

This study introduced a new connotation, i.e., environmentally sound invention (ESI), as a “bridge” between the driving force of environmentally sound technologies (ESTs) R&D activities and eco-innovation/green capabilities. The study investigates the COVID-19 pandemic's impact on ESI efficiency using a multi-methods model in three stages.

First, this study finds that the COVID-19 pandemic has resulted in a more challenging environment for ESI efficiency from 2020 to 2021. These results basically support HYPOTHESIS 1: The COVID-19 pandemic has a negative effect on regions' ESI efficiency. However, the negative effect of the pandemic is a decrease in the second year. These results coincide with indirect evidence from previous studies. On the one hand, the pandemic results in an increase in the driving force of ESTs' R&D activities including demand-pull [e.g., environmental protection awareness and sustainable consumption (40)], technology-push [e.g., environmental pollutant treatment technology (36)], and institution regulation [e.g., altering geopolitical and socio-economic norms (61)]. On the other hand, the pandemic results in a decrease in the output of the eco-innovation [e.g., (43)] and green capabilities [e.g., (62)]. To sum up, the efficiency is reduced because of the increase in input and the decrease in output.

Second, this study finds that there is a spatial effect on the relationship between the COVID-19 pandemic and ESI efficiency. These results support HYPOTHESIS 2: The COVID-19 pandemic has a significant spatial effect on ESI efficiency. Additionally, the results of this study show that external environmental factors significantly affect the slack term of ESI efficiency, that is, the potential increase in output. That means the ESI efficiency can be improved in a suitable environment (63). These results have two inspirations: how to create a better production environment for ESI and how to improve the environmental adaptability of regional ESI. This study argues that a common solution to these two issues is strengthening regional innovation cooperation (64). The ESI patent data collected by this research show that the main form of cooperation in China's ESI is FUCs. However, the two sides of the cooperation are often firms and universities in the same province. The number of green invention patents from FUCs accounted for 19% of the total from 2013 to 2021. The proportion of the green invention patent from the cooperation between provinces is much lower than this. For example, from 2013 to 2021, the proportion of the green invention patent that came from the cooperation between Beijing and other provinces was < 0.3% of all green invention patents in Beijing. Previous studies have proved the beneficial of collaborations between regions to ESTs' innovation (65). So we suggest that ESI activities should break the barrier of the border between regions to enhance the robustness of the whole system (66).

Based on these results, the theoretical implication of this study can be summarized as follows.

First, a new connotation, ESI, is introduced in this study. This new connotation may fill in gaps between the driving force and eco-innovation. There are several benefits to embedding the ESI in the process from driving force to eco-innovation. On the one hand, ESI can be beneficial to classify the type of eco-innovation. Schumpeter (67) classified five types of innovation from the perspective of “new combination”. There are also various types of eco-innovation. For example, Rennings (68) classified eco-innovations nature as technological, organizational, social, and institutional innovation. So “studies from the driving force to eco-innovation” is in a broad sense. A mediated indicator of factors such as ESI can make the theoretical path clearer. On the other hand, the concept of ESI can be beneficial to alleviate the indicator confusion problem in eco-innovation studies. Eco-innovation is not only a result but also a process, which makes the indicator of eco-innovation can be confusion (8). Some studies evaluate eco-innovation use indicators before innovation's market entry such as R&D (69), EST (70), or patents (71), some other studies evaluate eco-innovation use indicators after innovation's commercialization such as quality certifications (72). That may make a large measuring error using different indicators to evaluate the same connotation (73). Therefore, the measure of the ESI is a more appropriate way to evaluate eco-innovation from different perspectives.

Second, this study's results enrich the knowledge of the relationship between emergencies such as the pandemic and sustainable development of cleaner production. Previous studies have constructed two controversial theoretical paths of the relationship between emergencies and technological innovation in a broad sense. One of them takes an emergency as an “opportunity” for related development (74). The other one takes an emergency as “damage” for related development (33). This study enriches the knowledge of this relationship by providing new empirical evidence.

Third, for the change of ESI efficiency, is the pandemic a “crisis” or an “opportunity”? It's about time. The results of this study show that the negative effect of the pandemic is breaking out in 2020 and decreasing in 2021. Specifically, in the short term, the potential of the ESI increasing is reduced because of the pandemic. Previous studies also argue that innovating in response to the crisis, time seems crucial (75). Ebersberger and Kuckertz (76) recognized that start-ups have a short response time to the pandemic compared with universities. In the short-term, the pandemic can lead to increased input of the ESI innovation because of the pandemic's effect on the awareness of environmental protection and sustainable consumption (40), the increasing investment in environmental pollutant treatment technology (36), and the increasing investment on the digital economics for the sustainable development (39). However, it takes time to get the rewards of the increased input (77). Based on this result, we argue that the pandemic can be a “crisis” in the short term but an “opportunity” in the long term.

## 6. Conclusions and practical implications

### 6.1. Conclusions

This is the first study to investigate the COVID-19 pandemic's impact on ESI efficiency. The theoretical background from the driving force to eco-innovation with the mediated of ESI is sorted out. The empirical study is divided into three stages and conducted step-by-step tests using a multi-method model, including SBM efficiency measurement, spatial Tobit regression, SFA model, and three-stage DEA analysis.

First, the efficiency measure results indicate a decrease in the ESI efficiency after the outbreak of the pandemic. The results of the spatial Tobit model further show that the COVID-19 pandemic in 2020 harms the efficiency of green technological innovation in both 2020 and 2021. The results of the SFA model show that the direct and spatial effect is different in different years and for different inventors. These results support our assumptions that the ESI efficiency is negatively affected by the COVID-19 pandemic, and that the spatial effect exists.

Second, additional implications can be drawn from the empirical results. We compared the original and environmentally adjusted efficiency scores in 2020 and 2021. It can be found that the average efficiency rank of HH and LH provinces is more affected than in other areas in 2020. And combined with some other studies' results which have been referred in the discussion section, we argue that the pandemic can be a “crisis” in the short term but an “opportunity” in the long term.

Third, this study's results provide a new model to evaluate the influence of exogenous shocks to the process of eco-innovation. And the connotation of ESI makes the skeleton of the theoretical system from the driving force of EST R&D activities to eco-innovation/green capabilities clearer.

### 6.2. Practical implications

Based on the analysis of this research, the ESI efficiency under and post the COVID-19 pandemic is a complex system problem. The influence can be direct and indirect, on both input and output, on both regional macro and micro levels, and both public ESI innovation and private ESI innovation. On the base of these results, the practical implications and policy suggestions can be drawn as follows.

First, policymakers can try to learn from provinces that keep a high ESI efficiency with the high severity of the pandemic. This study's analysis identified the high and low-efficiency regions in China, the method is suitable for other countries or regions. For example, HH provinces such as Jiangsu, HL provinces such as Beijing are efficient provinces during 2020–2021. The experience can be found in other documents such as the system of environmental regulation policies (78) and recovery strategies (79).

Second, through the experience of provinces that have low severity of the pandemic, policies makers could learn how to decrease the damage of the pandemic to the ESI efficiency from other regions. For example, Zhejiang has a high severity in 2020 and low severity in 2021, the efficiency value is 1 no matter whether exclude the effect of the pandemic. Previous research has found that the institutional innovation in Zhejiang which emerged against the COVID-19 pandemic offers a way to transform the crisis into an opportunity (80).

## 7. Limitations and future research

There are also some important limitations of this study that need to be considered. First, this study uses a small sample for the investigation because of the data limitation. It may meet the minimum sample size for the regression, but the results' reliability is relatively not perfect. Since the pandemic is still affecting the world, further empirical research needs to be done in the future. Second, although we considered representative indicators as control variables based on classical driving force theories of green innovation. Many other factors can influence ESI and its efficiency. However, because of the data limitation and multicollinearity problems, we cannot account for other indicators in the regression model. That may result in problems of missing variables.

## Data availability statement

The raw data supporting the conclusions of this article will be made available by the authors, without undue reservation.

## Author contributions

XW, RL, and ZL contributed to conception and design of the study. XW organized the database, performed the statistical analysis, and wrote the first draft of the manuscript. RL and ZL wrote sections of the manuscript. All authors contributed to manuscript revision, read, and approved the submitted version.
